# Targeted Exome Sequencing Provided Comprehensive Genetic Diagnosis of Congenital Anomalies of the Kidney and Urinary Tract

**DOI:** 10.3390/jcm9030751

**Published:** 2020-03-10

**Authors:** Yo Han Ahn, Chung Lee, Nayoung K. D. Kim, Eujin Park, Hee Gyung Kang, Il-Soo Ha, Woong-Yang Park, Hae Il Cheong

**Affiliations:** 1 Department of Pediatrics, Seoul National University College of Medicine, Seoul 03080, Korea; medicalpooh@snu.ac.kr (Y.H.A.); eujinpark@hallym.or.kr (E.P.); ilsooha@snu.ac.kr (I.-S.H.); cheonghi@snu.ac.kr (H.I.C.); 2 Department of Pediatrics, Seoul National University Children’s Hospital, Seoul 03080, Korea; 3 Samsung Genome Institute, Samsung Medical Center, Seoul 06351, Korea; chung.lee@kr-geninus.com (C.L.); bionkdk@gmail.com (N.K.D.K.); woongyang.park@samsung.com (W.-Y.P.); 4 Department of Health Sciences and Technology, Samsung Advanced Institute for Health Sciences and Technology, Sungkyunkwan University, Seoul 06351, Korea; 5 Department of Pediatrics, Kangnam Sacred Heart Hospital, Hallym University College of Medicine, Seoul 07441, Korea; 6 Kidney Research Institute, Medical Research Center, Seoul National University College of Medicine, Seoul 03080, Korea; 7 Department of Molecular Cell Biology, Sungkyunkwan University School of Medicine, Suwon 16419, Korea

**Keywords:** congenital anomalies of kidney and urinary tract, genetic analysis, single nucleotide variant, copy number variant

## Abstract

Congenital anomalies of the kidney and urinary tract (CAKUT) are the most common cause of chronic kidney disease in children. The search for genetic causes of CAKUT has led to genetic diagnosis in approximately 5–20 % of CAKUT patients from Western countries. In this study, genetic causes of CAKUT in Korean children were sought using targeted exome sequencing (TES) of 60 genes reported to cause CAKUT in human or murine models. We identified genetic causes in 13.8% of the 94 recruited patients. Pathogenic single nucleotide variants of five known disease-causing genes, *HNF1B*, *PAX2*, *EYA1*, *UPK3A*, and *FRAS1* were found in 7 cases. Pathogenic copy number variations of 6 patients were found in *HNF1B*, *EYA1*, and *CHD1L*. Genetic abnormality types did not significantly differ according to CAKUT phenotypes. Patients with pathogenic variants of targeted genes had syndromic features more frequently than those without (*p* < 0.001). This is the first genetic analysis study of Korean patients with CAKUT. Only one-seventh of patients were found to have pathogenic mutations in known CAKUT-related genes, indicating that there are more CAKUT-causing genes or environmental factors to discover.

## 1. Introduction

Congenital anomalies of the kidney and urinary tract (CAKUT) are the most common cause of chronic kidney disease (CKD) in children, accounting for two-thirds of pediatric CKD [1,2]. CAKUT represent any abnormalities in number, size, shape, or anatomic position of the kidneys or other parts of the urinary tract, such as renal agenesis, renal hypodysplasia, multicystic dysplastic kidney, vesicoureteral reflux, ureteropelvic junction obstruction, ureterovesical junction obstruction, and posterior urethral valves. While some CAKUT, such as isolated vesicoureteral reflux or other primary urinary tract anomalies, do not impair renal function, others with a primary defect in kidney parenchymal development often progress to CKD. In addition, patients with CAKUT often have extrarenal manifestations as well, such as hearing loss, neurocognitive disorders, and cardiac anomalies, which affect growth and development. Therefore, many children with CKD resulting from CAKUT experience extrarenal manifestations besides the long-standing CKD, which may also lead to complications such as impairments in physical and psychosocial development [3,4,5] as well as high morbidity and mortality.

Disruption of renal development, caused by environmental factors or the dysfunction of genes involved in this process, can lead to CAKUT [6]. Genetic causes of CAKUT have been revealed since 1995 when a mutation in *PAX2* was first discovered to cause optic nerve coloboma, renal hypodysplasia, and vesicoureteral reflux [7]. Previous studies have identified over 40 genomic disorders and more than 50 genes have been reported to be associated with CAKUT [8,9]. Currently, up to 18% of patients with CAKUT can be explained by monogenic causes, most of which have a dominant pattern of inheritance [9,10]. Moreover, three large cohort studies using chromosomal microarrays identified that 4.5–16.6% of patients with CAKUT harbor genomic imbalances, especially in patients with renal hypodysplasia [11,12,13]. Most copy number variations (CNVs) causing CAKUT have been previously reported to be associated with other developmental disorders, such as developmental delay, neurocognitive disorders, and cardiac malformations [8].

Genetic diagnosis of CAKUT can help physicians correctly diagnose the extent of the problem and evaluate patients for extrarenal manifestations, in addition to enabling family evaluation and genetic counseling. However, it remains challenging because of genetic and phenotypic heterogeneity and incomplete penetrance of CAKUT. In the literature, diagnostic yield of genetic testing of patients with CAKUT is approximately 5–20%, representing either single nucleotide variants (SNVs) or CNVs of the relevant genes [11,12,13,14,15,16,17,18,19]. Most of the large studies on CAKUT are from Western countries, therefore genetic background of CAKUT in Asian populations is yet to be elaborated [20,21]. Moreover, optimal and cost-effective method of genetic diagnosis in CAKUT that can detect both SNV and CNV still needs to be found.

In this study, we aimed to elucidate the genetic causes of CAKUT in Korean children using targeted exome sequencing (TES) of CAKUT-related genes.

## 2. Methods

### 2.1. Study Participants

Patients with CAKUT were recruited from the pediatric nephrology clinic at Seoul National University Children’s Hospital. All patients were diagnosed with CAKUT on the basis of renal imaging studies. Inclusion criteria were renal hypodysplasia, renal agenesis, multicystic dysplastic kidney, bilateral vesicoureteral reflux, and bilateral obstructive uropathy (ureteropelvic junction obstruction, ureterovesical junction obstruction, or posterior urethral valves). Syndromic CAKUT was defined as conditions that are associated with other congenital anomalies, such as renal cysts and diabetes (RCAD) syndrome, branchio-oto-renal (BOR) syndrome, renal coloboma syndrome, and Fraser syndrome, as previously described [8]. This study was approved by the Institutional Review Board of Seoul National University Hospital. Informed consent was obtained from all individual participants and/or their parents.

### 2.2. Targeted Exome Sequencing and Bioinformatics Analysis

**Figure 1:**
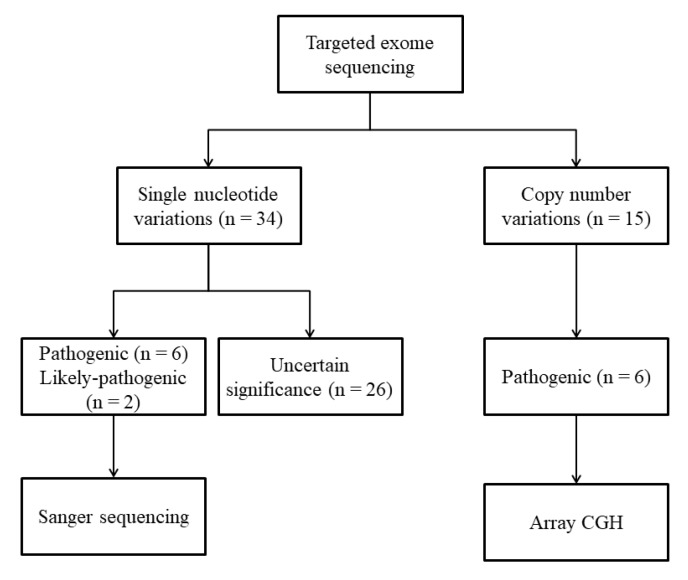
Schematic workflow for the identification of single nucleotide variants and copy number variants.

Through a literature review, 60 genes that had been reported to cause CAKUT in humans or murine models were selected (Table S1). We designed baits covering all exon regions of the targeted 60 genes (Twist Custom Panels; Twist Bioscience, San Francisco, CA). Genomic DNA was extracted from whole blood and sequencing libraries were prepared using Twist modular library preparation kits. Targeted sequencing was performed with 2x 101 bp paired-end reads on an Illumina MiSeq platform (Illumina, San Diego, CA). Bioinformatics analyses using sequenced reads were performed as previously described [22,23,24,25]. Variants were annotated with various information using ANNOVAR (Annotate Variation) [26] utilizing (i) population databases such as 1000 genome phase III, ExAC (Exome Aggregation Consortium), and KRGDB (Korean Reference Genome Database)(http://coda.nih.go.kr/coda/KRGDB/), (ii) disease databases such as OMIM (Online Mendelian Inheritance in Man) and sequencing databases such as RefSeqGene, and (iii) in silico predictive algorithms such as FATHMM (Functional Analysis through Hidden Markov Models), MutationAssessor, MutationTaster, SIFT, Polyphen, GERP, and Phylop for interpretation and classification of variants according to the American College of Medical Genetics and Genomics (ACMG) guidelines [27]. Workflow of prioritization and filtering the annotated variants is shown in Table S2. Variants classified as pathogenic or likely pathogenic based on ACMG guidelines were confirmed by Sanger sequencing (Figure 1). CNVs were calculated using aligned read counts in target regions by an in-house relative comparison method. Interpretation of detected CNVs was based on size, gene content, and overlap with known disease-associated regions according to the ACMG guidelines for postnatal CNV calling [28]. Classified pathogenic CNVs were re-confirmed by array comparative genomic hybridization (array CGH; Cytoscan 750k array; Affymetrix, Santa Clara, CA). Whenever possible, genotypes of relevant genes were obtained from the parents.

### 2.3. Statistical Analysis

To determine statistical differences between groups with or without pathogenic variants, categorical variables were analyzed using the chi-square test or Fisher’s exact test and continuous variables were compared using the *t*-test or Mann-Whitney U test. All values were reported as a median (interquartile range; IQR). Kaplan-Meier analysis was used to assess renal survival. *p* values < 0.05 were considered statistically significant. Statistical analysis was performed using SPSS version 23.0 (IBM, Armonk, NY, USA).

## 3. Results

A total of 94 unrelated Korean patients (M:F 78:16) with CAKUT were recruited (Table S4). The most common phenotype of CAKUT was bilateral renal hypodysplasia (n = 44), followed by unilateral renal hypodysplasia/renal agenesis/multicystic dysplastic kidney with contralateral other anomalies (n = 27), bilateral vesicoureteral reflux (n = 14), unilateral renal hypodysplasia/renal agenesis/multicystic dysplastic kidney (n = 5), and bilateral obstructive uropathy (n = 4). A renal anomaly of each kidney was counted independently as a single unit. Among the 188 renal units of 94 patients, the most common anomaly was renal hypodysplasia (n = 111), followed by vesicoureteral reflux (n = 69), obstructive uropathy (n = 10), multicystic dysplastic kidney (n = 10), renal agenesis (n = 9), and hydronephrosis (n = 4) (Table S4).

Five patients had a family history of CAKUT, of which two had siblings with CAKUT and four had family members with CAKUT in previous generations (mother and grandparents). Sixty-two patients had extrarenal manifestations, such as perinatal problems including prematurity, oligohydroamnios, and intrauterine growth retardation (n = 31), neurodevelopmental disorders (n = 16), cardiac disease (n = 10), genital anomalies (n = 9), eye diseases (n = 8), hearing loss (n = 8), diabetes (n = 6), and hypothyroidism (n = 5) (Table S4). Eighty-eight patient (93.6%) exhibited impaired renal function (defined as estimated glomerular filtration rate < 90 mL/min/1.73 m^2^) at their last visit and 40 patients (42.6%) developed end stage renal disease (ESRD) at the median age of 13.8 (IQR, 8.9–19.8) years.

### 3.1. Single Nucleotide Variants

We identified pathogenic or likely pathogenic SNVs of five known disease-causing genes in seven cases of *HNF1B* (n = 2), *PAX2* (n = 2), *EYA1* (n = 1), *UPK3A* (n = 1), and *FRAS1* (n = 1; compound heterozygous) (Table 1). These genes are known to have autosomal dominant inheritance, except for *FRAS1*, which exhibits autosomal recessive inheritance [9]. The inheritance pattern was confirmed in one patient (ID 29), who inherited his *HNF1B* mutation from his father; however, the father has no kidney problems or extrarenal manifestations, while the patient’s grandfather had juvenile-onset diabetes mellitus (genetic test not available). In two patients (ID 92 and 95), variants were confirmed as de novo after confirming the absence of the variants in their parents. Five SNVs in genes with autosomal dominant inheritance were frameshift or stop-gain mutations, suggesting loss of function. These truncating mutations were classified as pathogenic SNVs based on the absence in the population databases, in silico prediction of deleterious effect, and patients’ clinical phenotypes (Table 1). Pathogenic or likely-pathogenic variants identified in the patients and their parents (when available) were confirmed by Sanger sequencing. The rest of the twenty-six missense mutations of 21 patients in known CAKUT-causing genes were classified as variants of uncertain significance (Table S3).

Renal phenotypes of mutations in *HNF1B*, *PAX2*, and *EYA1* were bilateral renal hypodysplasia or unilateral renal hypodysplasia with contralateral multicystic dysplastic kidney. Two patients with bilateral renal hypodysplasia and optic nerve anomalies carried variants in *PAX2* (p.Arg115Pro and p.Val26Glyfs*28, respectively), causing renal coloboma syndrome. A missense mutation at Arg-115 in the *PAX2* gene was determined as a likely pathogenic variant based on its absence in the population data, a deleterious effect predicted by in silico analysis, and the compatible phenotype of the patient. A patient carrying a novel truncating variant (p.Gly563*) in *EYA1*, which is known to cause BOR, had bilateral renal hypodysplasia and underwent excision of branchial cleft cyst at the age of 11.8 years. This patient was born prematurely with small sized kidneys and oligohydramnios noted before birth, and developed lymphoma at the age of 2.7 years.

A truncating mutation of *UPK3A* (p.Leu156Valfs*85) was expressed as bilateral vesicoureteral reflux with stage 2 CKD at the age of 4.0 years. A patient with right renal agenesis and left renal cysts carried compound heterozygous mutations in *FRAS1* (p.Tyr2273* from the father and p.Gly3456Asp from the mother). Her older brother who had left MCDK, right ureteropelvic junction obstruction, and posterior urethral valves developed stage 3 CKD at the age of 18.8 years (genetic test not available).

All patients with pathogenic or likely pathogenic SNVs developed CKD during childhood.

### 3.2. Copy Number Variants

Six patients showed pathogenic CNVs (deletions in 4 and duplications in 2) in the following targeted genes, *HNF1B* (n = 4), *EYA1* (n = 1), and *CHD1L* (n = 1) (Table 2). The size of the rearrangements ranged from 1.48 to 2.20 Mb.

Four patients had a deletion or duplication of chromosome 17q12 containing *HNF1B* with variable renal manifestations including renal hypodysplasia, multicystic dysplastic kidney, vesicoureteral reflux, ureteral obstruction, and renal cortical cysts. All patients had variable extrarenal manifestations including diabetes mellitus, choledochal cyst, hypomagnesemia, hyperuricemia, and epilepsy. Among the four patients with a CNV of chromosome 17q12, one patient with a duplication (ID 82) had normal renal function at last follow up at 18.1 years, while the others with deletions developed CKD during childhood. Two patients with the 17q12 deletion (ID 50 and 82) were diagnosed with diabetes at the age of 23.7 and 18.1 years, respectively, while another patient with the same deletion (ID 34) had not developed diabetes at last follow up at 13.6 years. Regarding extrarenal symptoms other than diabetes, a patient with a 17q12 duplication (ID 58) complained of multiple neuropathic pain, weakness, and tremors after renal transplantation at the age of 21.1 years and was suspected to have parietal lobe epilepsy or psychiatric disorders. Patient ID 34 with a 17q12 deletion had multiple congenital anomalies including imperforate anus, hypospadias, and choledochal cysts. He had undergone several surgeries for these anomalies and experienced operation-related complications.

A patient with a deletion of 8q13.3 containing *EYA1* (ID 15) had bilateral renal hypodysplasia with pre-auricular pits and severe hearing loss requiring hearing aids from the age of 6.0 years. One patient with bilateral renal cortical cysts and renal stones (ID 41) had a duplication of 1q21.1 encompassing *CHD1L*. His extrarenal manifestation was patent ductus arteriosus, which required device closure at the age of 7.6 years.

### 3.3. Genotype and Phenotype Correlations

**Figure 2:**
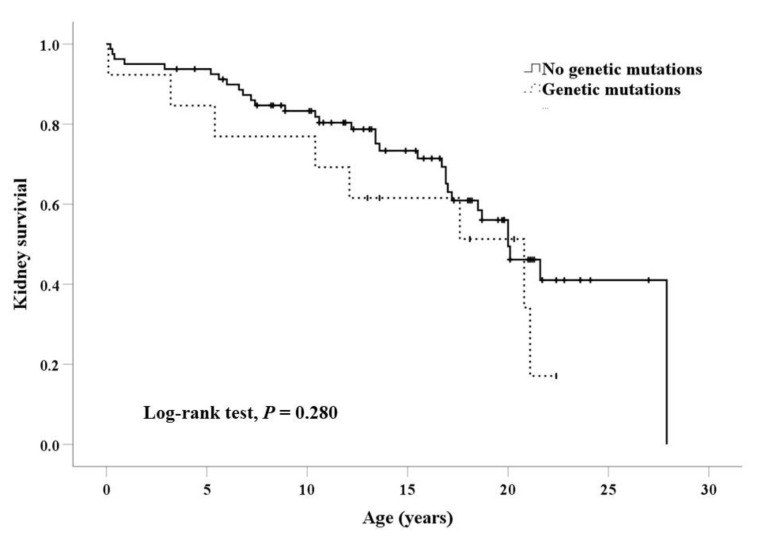
Kidney survival according to the presence of pathogenic variants.

The types of genetic abnormalities did not significantly differ according to CAKUT phenotypes. Pathogenic variants were identified in three of nine patients with unilateral multicystic dysplastic kidney and other contralateral anomalies, with a relatively high detection rate. Bilateral anomalies of kidneys were more frequent in patients with pathogenic variants (76.9%) than those without pathogenic variants (51.9%), but the difference was not statistically significant (*p* = 0.091). Patients with pathogenic variants had syndromic features more frequently (84.6%) than those without variants (25.9%; *p* < 0.001; Table 3). Family history of CAKUT was rare in both groups with or without mutations. Perinatal problems also did not differ between the two groups. Kaplan–Meier survival curves showed that the presence of genetic mutations did not affect renal survival (Log-rank test, *p* = 0.280; Figure 2).

## 4. Discussion

This is the first report on the genetic diagnosis in a Korean patient cohort of CAKUT, which has long-term follow up data on renal outcome. Using TES, our study identified pathogenic SNVs and CNVs in 7.4% and 6.4% of patients with CAKUT, respectively. The detection rate of genetic causes in CAKUT is lower than that of other causes of early-onset CKD, partially because genetic analysis of CAKUT is difficult due to genetic heterogeneity, incomplete penetrance, and variable expressivity [9,31]. The genetic diagnosis rate of 13.8% is similar to previous studies employing various detection methods. TES achieved a 1–6% positive result in patients with several types of CAKUT [16,17], while whole exome sequencing (WES) studies found causative mutations of known CAKUT genes in 11–14% of patients [14,15,21] and mutations of novel genes in 8% of families [14]. Previous CNV studies reported that 4.5–16.6% of patients with variable CAKUT were carrying pathogenic CNVs [11,12,13,18,19]. While most previous studies tried to identify only SNVs or CNVs, we hypothesized that simultaneous evaluation of pathogenic SNVs and CNVs using TES would increase the overall diagnostic yield. Although we achieved a better diagnostic yield than did other studies using TES, the yield was not higher than that of other studies using WES. Another study that had used such an integrated approach employed WES and evaluated not only SNVs and but also CNVs using two CNV detection tools [15]. Therefore, we showed that incorporation of CNV evaluation is possible as well as helpful for the genetic diagnosis of CAKUT via TES. In addition, the number of targeted genes in a gene panel is also important for the detection rate; while previous reports tested 208 and 17 genes, our gene panel included 60 genes, though the detection rate of SNVs is similar compared to previous studies that employed TES [16,17]. Differences in inclusion/exclusion criteria of subjects might also have affected the detection rate of causative genetic variants in a disease population. Selectively testing severe and syndromic CAKUT cases or patients from consanguineous families would result in a higher detection rate compared to testing a general cohort of CAKUT encompassing the whole spectrum of all CAKUT phenotypes. To increase diagnostic yield, our study included only patients with severe CAKUT phenotypes, such as renal agenesis, renal hypodysplasia, and cystic dysplasia, which are expected to profoundly impair long-term renal survival [12,32,33]. In this study, 93.6% of patients developed CKD during childhood. One pediatric CKD cohort study revealed that a subset of patients diagnosed with renal hypodysplasia was particularly enriched for known genomic disorders (10.5%) [12]. Another study demonstrated that stillborn fetuses with renal agenesis or severe dysplasia had a high positive rate (30%) of disease-causing mutations, although only *RET*, *GDNF*, and *GFRA1* genes were evaluated [33]. We theorized that genetic abnormalities would be detected more frequently in patients with severe forms of CAKUT than those with mild forms or unilateral anomalies. However, baseline clinical characteristics and renal survival did not differ regardless of the presence of genetic mutations, which is similar to what was previously reported [12,20].

In our study, mutations of the *HNF1B* gene were the most commonly identified genetic cause, accounting for 46.2% of patients with pathogenic variants. This result is in line with previous studies reporting that *HNF1B* mutations were the most prevalent in patients with CAKUT [11,12,17,34]. Moreover, the most common pathogenic CNVs of CAKUT are 17q12 deletions; the chromosome 17q12 region containing *HNF1B* is highly susceptible to genomic rearrangement by non-allelic homologous recombination between flanking segmental duplications [35,36]. *HNF1B* encodes hepatocyte nuclear factor 1-β, which plays a critical role in ureteric bud branching and renal tubular development in early nephrogenesis [37,38]. Renal malformations associated with *HNF1B* mutations vary and include renal cysts, renal hypodysplasia, renal agenesis, cystic renal dysplasia, and ureteral defects. Extrarenal features of *HNF1B* mutations include early-onset diabetes mellitus, pancreatic hypoplasia, developmental delay, genital tract malformations, abnormal liver function, hypomagnesemia, hyperuricemia, and early-onset gout. Therefore, *HNF1B*-associated disease is considered a multi-system disorder [39]. In this study, patients with *HNF1B* mutations also presented with variable, multi-systemic phenotypes. Interestingly, one patient with a 17q12 duplication manifested atypical symptoms of suspected neuropsychiatric disorders; indeed, patients with 17q12 deletions or duplications have been reported to have a broad range of psychiatric and neurologic features [40]. This may be a contiguous-gene syndrome, as the chromosome 17q12 region includes *LHX1* [11,12], which encodes LIM homeobox 1, a transcriptional factor that functions in the development of neural cells [41,42].

Pathogenic variants in *PAX2* and *EYA1* were common in our study, which is similar to previous findings [15,17]. Mutations in *PAX2* and *EYA1* cause the autosomal dominant disorders renal coloboma syndrome and BOR syndrome, respectively. Bower et al. [43] reported that 77% of 173 patients with *PAX2* mutations have ophthalmological abnormalities of the optic nerve, retina, macula, and lens. Meanwhile, *EYA1* mutations account for 30%–35% of BOR syndrome, which is characterized by branchial defects, malformations of the outer, middle, and inner ear associated with deafness, and renal anomalies [44,45]. In our study, patients with these genetic variants also presented with these characteristic clinical manifestations.

Compared with previous studies employing Caucasian patients with CAKUT, our study did not find pathogenic variants in *RORO2*, *SALL1*, *FREM2*, or *RET*, which were also often identified in previous studies [14,17,33,46]. Our findings are similar to a Japanese study, where *HNF1B* and *PAX2* were reported as the most common causative genes and none of the above four genes were found [20]. On the other hand, a Chinese study identified pathogenic variants in *HNF1B*, *UMOD*, *NEK8*, and *BBS2* genes [21]. Therefore, these difference may be associated with ethnic differences.

Our study revealed syndromic CAKUT had a higher rate of pathogenic variants than did isolated CAKUT (34.4% vs. 3.2%). In this regard, extrarenal manifestations may provide an important clue for uncovering the genetic cause of CAKUT. Some syndromic CAKUT encompass characteristic extrarenal manifestations, such as hearing loss and neck anomalies in BOR syndrome (*EYA1*), optic nerve coloboma in renal coloboma syndrome (*PAX2*), and hyperuricemia and diabetes in RCAD syndrome (*HNF1B*). Evaluation of patients with CAKUT should include screening for associated extrarenal manifestations and detailed history taking of the patient and family to help identify the underlying genetic cause. On the other hand, molecular diagnosis can help physicians screen and identify hidden or subtle clinical manifestations of other organs, which significantly affect the management and prognosis of patients. Some CAKUT-causing gene mutations are associated with the risk of developmental delay, learning disabilities, and other neuropsychiatric disorders that benefit from early detection and intervention. In this study, 4 of 13 (30.7%) patients with pathogenic variants had neurodevelopmental disorders. Some extrarenal manifestations, including hearing loss, diabetes, hyperuricemia, and infected branchial cleft cyst, may present later. If physicians are aware of the possible clinical features associated with genetic defects, targeted workup or surveillance based on current recommendations can be established [12]. Therefore, genetic analysis for CAKUT genes is highly recommended for CAKUT patients, especially with syndromic CAKUT.

In this study, only one-seventh of the patients obtained genetic diagnosis using TES. It suggests that other genes not included in our gene panel or non-genetic factors have contributed to the occurrence of CAKUT. Clearly, epigenetic and environmental factors would affect the development of CAKUT [6]; large studies revealed that CAKUT were associated with prenatal risk factors, such as gestational diabetes, maternal obesity, and low birth weight [47,48]. Marked variation of the clinical phenotype and severity of CAKUT among individuals carrying the same mutation also indicates that complex genetic or non-genetic mechanisms are involved in the pathogenesis of CAKUT. In addition, as suggested for other complex developmental traits such as cleft palate and congenital heart disease [49,50], the polygenic model might also be able to explain the vast heterogeneity of CAKUT.

There are several limitations associated with this study. First, while we assessed CNVs, only those involving captured areas were searched, as we employed TES instead of WES. Therefore, we may have missed variations in noncoding regions of tested genes, as well as those involving other regions that are not covered by TES. Due to the lack of resources, only patients with CNVs were assessed by array CGH (n = 6). Therefore, other subjects may also possess CNVs that were not detected because we did not perform array CGH for all subjects. Second, discovery of novel genes is not possible when employing TES instead of WES or whole-genome sequencing. With advances in technology, especially for the genetic diagnosis of such disease groups of CAKUT with genetic heterogeneity and multiple causative CNVs, whole-genome sequencing may be the better technique. Third, trio samples were not available for all patients, therefore assessment of penetrance or segregation was not sufficient. Lastly, functional studies of novel mutations were not performed. Nonetheless, this is the first report on the genetic diagnosis of CAKUT in Korean patients, and one of the few studies reporting data on Asia patients. In addition, our longitudinal outcome data on this unique Korean CAKUT patient cohort could bring new opportunities for future studies such as development of predictive models and further investigations of yet-to known genetic abnormalities including variants of uncertain significance.

## 5. Conclusions

This is the first genetic analysis study conducted on Korean patients with CAKUT using TES. Only one-seventh of the patients were found to have pathogenic mutations of known CAKUT-related genes, indicating that there are still more CAKUT-inducing genes or environmental factors to discover. Nonetheless, the identified mutations are important, enabling us to predict outcomes and provide proactive care and adequate genetic counseling for patients and families with CAKUT.

## Figures and Tables

**Table 1:** Pathogenic or likely pathogenic single nucleotide variants.

Patient ID	Sex/Age (Year)	Gene	Nucleotide	Amino Acid	Segregation	Frequency		Renal Phenotype	Extrarenal Phenotype	Renal Function	Ref	Classification
						KRGDB	ExAC					
29	M/5.3	*HNF1B*	c.541C>T	p.Arg181*	Father	0	0	Bilateral RHD and renal cysts	Preterm, oligohydramnios, inguinal hernia, and developmental delay	ESRD at 1 mo	[29]	Pathogenic
92	M/10.4	*HNF1B*	c.1103_1116del	p.His368Argfs*27	De novo	0	0	Left MCDK and right RHD with renal cysts	Oligohydramnios, choledochal cyst, and hypomagnesemia	CKD	No	Pathogenic
07	M/18.4	*PAX2*	c.344G>C	p.Arg115Pro	NA	0	0	Bilateral RHD	Morning glory optic discs, dilated cardiomyopathy, and hypothyroidism	ESRD at 10.4 yr	No	Likely pathogenic
95	M/14.5	*PAX2*	c.76dupG	p.Val26Glyfs*28	De novo	0	0	Bilateral RHD and renal cysts	Optic nerve anomaly, hemolytic anemia, and ADHD	ESRD at 3.2 yr	No	Pathogenic
35	M/10.9	*EYA1*	c.1582G>T	p.Gly563*	NA	0	0	Bilateral RHD	Preterm, oligohydramnios, branchial cleft cyst, lymphoma, developmental delay, autism, and inguinal hernia	ESRD at 12.1 yr	No	Pathogenic
55	M/19.9	*UPK3A*	c.466_467del	p.Leu156Valfs*85	NA	0	0.0004	Bilateral VUR	No	CKD	No	Pathogenic
1	F/21.1	*FRAS1*	c.6819T>A c.10367G>A	p.Tyr2273* p.Gly3456Asp	Father Mother	0.0005 0	0.00002 0	Right renal agenesis and left renal cysts	No	ESRD at 20.8 yr	No No	Pathogenic Likely pathogenic

Transcript number; *HNF1B*, NM_000458.4; *PAX2*, NM_000278.5; *EYA1*, NM_000503.6; *UPK3A*, NM_006953.4; *FRAS1*, NM_025074.7. Yr, years; KRGDB, Korean Reference Genome Database; ExAC, Exome Aggregation Consortium; Ref, reference; RHD, renal hypodysplasia; mo, months; ESRD, end stage renal disease; MCDK, multicystic dysplastic kidney; CKD, chronic kidney disease; NA, not available; ADHD, attention deficit hyperactivity disorder; VUR, vesicoureteral reflux.

**Table 2:** Pathogenic copy number variants.

PatientID	Sex/Age (Year)	Chromosomal Region	CNV Type	Start (Mb)	End (Mb)	Size (Mb)	Involved OMIM Genes	Renal Phenotype	Extrarenal Phenotype	Renal Function	Reference
34	M/12.7	17q12	Del	34.82	36.38	1.56	*ZNHIT3*, *MYO19*, *PIGW*, *GGNBP2*, *DHRS11*, *MRM1*, *LHX1*, *AATF*, *ACACA*, *TADA2A*, *DUSP14*, *SYNRG*, *DDX52*, *HNF1B*, and *TBC1D3*	Bilateral RHD with renal cysts	Preterm, imperforate anus, hypospadias, and choledochal cyst	CKD	[12]
50	M/26.1	17q12	Del	34.47	36.24	1.76	*TBC1D3B*, *CCL3L3*, *CCL4L2*, *TBC1D3C*, *CCL3L1*, *TBC1D3H*, *TBC1D3G*, *ZNHIT3*, *MYO19*, *PIGW*, *GGNBP2*, *DHRS11*, *MRM1*, *LHX1*, *AATF*, *ACACA*, *TADA2A*, *DUSP14*, *SYNRG*, *DDX52*, and *HNF1B*	Left MCDK and right mid-ureteral obstruction	Post-transplant diabetes mellitus	ESRD at 17.6 yr	[12]
58	M/22.8	17q12	Dup	34.82	36.37	1.55	*ZNHIT3*, *MYO19*, *PIGW*, *GGNBP2*, *DHRS11*, *MRM1*, *LHX1*, *AATF*, *ACACA*, *TADA2A*, *DUSP14*, *SYNRG*, *DDX52*, *HNF1B*, and *TBC1D3*	Bilateral VUR and right MCDK	Parietal lobe epilepsy, multiple neuropathic pain, hypomagnesemia, and hyperuricemia	ESRD at 21.1 yr	[12]
82	M/15.5	17q12	Del	34.82	36.30	1.48	*ZNHIT3*, *MYO19*, *PIGW*, *GGNBP2*, *DHRS11*, *MRM1*, *LHX1*, *AATF*, *ACACA*, *TADA2A*, *DUSP14*, *SYNRG*, *DDX52*, and *HNF1B*	Bilateral RHD with renal cysts	Diabetes mellitus	Normal	[12]
15	F/14.5	8q13.3	Del	71.94	74.15	2.20	*EYA1*, *MSC*, *TRPA1*, *KCNB2*, and *TERF1*	Bilateral RHD	Pre-auricular pit and hearing loss	ESRD at 5.4 yr	[30]
41	M/18.1	1q21.1	Dup	146.00	147.99	1.99	*NBPF12*, *PRKAB2*, *FMO5*, *CHD1L*, *BCL9*, *ACP6*, *GJA5*, *GJA8*, *GPR89B*, and *NBPF11*	Bilateral RHD with renal cysts and renal stone	Patent ductus arteriosus, pancreas hypoplasia, common bile-duct dilatation, and hepatic cyst	CKD	[19]

Yr, years; CNV, copy number variant; OMIM, Online Mendelian Inheritance in Man; Del, deletion; RHD, renal hypodysplasia; CKD, chronic kidney disease; MCDK, multicystic dysplastic kidney; ESRD, end stage renal disease; Dup, duplication; VUR, vesicoureteral reflux; NPHP, nephronophthisis.

**Table 3:** Comparison between patients with and without pathogenic/likely pathogenic variants.

Characteristic	Pathogenic or Likely Pathogenic Variants		*p* Value
	Positive (n = 13)	Negative (n = 81)	
Male sex	11 (84.6)	67 (82.7)	1.000
Age at enrollment, years	15.5 (12.2–19.9)	15.3 (9.3–19.3)	0.360
Age at last follow up, years	18.1 (13.8–22.4)	17.2 (11.9–21.3)	0.324
Renal phenotype			0.293
Bilateral lesions			
bRHD with/without Others ^a^	8 (61.5)	36 (44.4)	
uAgenesis + cOthers ^a^	1 (7.7)	6 (7.4)	
uMCDK + cOthers ^a^	3 (23.1)	6 (7.4)	
uRHD + cOthers ^a^	0	11 (13.6)	
bVUR	1 (7.7)	13 (16.0)	
bObstructive uropathy ^b^	0	4 (4.9)	
Unilateral lesions	0	5 (6.2)	
Bilateral renal anomalies	10 (76.9)	42 (51.9)	0.091
Kidney function			0.282
Normal	1 (7.7)	5 (6.2)	
CKD	4 (30.8)	44 (54.3)	
ESRD	8 (61.5)	32 (39.5)	
Age at diagnosis of ESRD, years	13.6 (10.4–20.3)	13.9 (8.9–19.7)	0.914
Age at 50% kidney survival ^c^, years	20.8 (10.2–31.4)	20.0 (17.2–22.8)	0.280
Family history of CAKUT ^d^	1 (7.7)	4 (4.9)	0.533
Syndromic CAKUT	11 (84.6)	21 (25.9)	<0.001
Premature birth	3 (23.1)	20 (24.7)	1.000
Small for gestational age	3 (23.1)	14 (17.3)	0.698
Oligohydramnios	3 (23.1)	19 (23.5)	1.000

Values are expressed as numbers (%) and median (interquartile range). ^a^ Including renal hypodysplasia, multicystic dysplastic kidney, and renal agenesis. ^b^ Including posterior urethral valve (n = 3) and bilateral ureteropelvic junction obstruction (n = 1). ^c^ The median (95% confidence interval) estimated by Kaplan–Meier survival analysis. ^d^ Reporting by the patients and parents. b, bilateral; u, unilateral; c, contralateral; RHD, renal hypodysplasia; VUR, vesicoureteral reflux; MCDK, multicystic dysplastic kidney; CKD, chronic kidney disease; ESRD, end stage renal disease; CAKUT, congenital anomalies of the kidney and urinary tract.

## Supplementary Materials

The following are available online at https://www.mdpi.com/2077-0383/9/3/751/s1, Table S1: The 60 genes included in the gene panel design, Table S2: Single nucleotide variant prioritization and filtering workflow, Table S3: Variants of uncertain significance, Table S4: Clinical presentation of study participants.
