# Supplementary material for: Targeted Exome Sequencing Provided Comprehensive Genetic Diagnosis of Congenital Anomalies of the Kidney and Urinary Tract
Source: J Clin Med. 2020 Mar 10;9(3):751. doi: 10.3390/jcm9030751 (PMC7141392; doi:10.3390/jcm9030751)
Supplement: Supplementary file 1 [file jcm-09-00751-s001.pdf]

Supplementary Material: Targeted Exome Sequencing Provided Comprehensive Genetic Diagnosis of Congenital Anomalies of the Kidney and Urinary Tract

Yo Han Ahn [1,2,†], Chung Lee [3,4,†], Nayoung K. D. Kim [3], Eujin Park [1,5], Hee Gyung Kang [1,2,6,*], Il-Soo Ha [1,2,6], Woong-Yang Park [3,4,7] and Hae Il Cheong [1,2,6]

S. Table 1. The 60 genes included in the gene panel design.

Gene	MIM gene ID	Inheritance	Renal phenotype	Reference
ACE	106180	AR	Renal tubular dysgenesis	[1]
ACVR2B	602730	Unknown	Renal agenesis and renal hypoplasia in mice	[2]
AGT	106150	AR	Renal tubular dysgenesis	[1]
AGTR1	106165	AR	Renal tubular dysgenesis	[1,3]
AGTR2	300034	AR	UPJO	[4]
BICC1	614295	AD	Cystic renal dysplasia	[5]
BMP4	112262	AD	Renal hypodysplasia	[6]
BMP7	112267	AD	Renal dysplasia	[7,8]
CDC5L	602868	AD	Multicystic renal dysplasia	[8,9]
CHD1L	613039	AD	Renal hypodysplasia, VUR, and UPJO	[8,10]
DLX5	600028	AR	Urethral malformation in mice	[11]
DLX6	600030	Unknown	Urethral malformation in mice	[11]
DSTYK	612666	AD	Renal hypodysplasia and UPJO	[12]
EMX2	600035	Unknown	Urinary tract anomalies in mice compound heterozygous for Pax2 and Emx2	[13]
EYA1	601653	AD	Branchio-oto-renal syndrome, multicystic dysplastic kidney, and renal hypoplasia	[14,15]
FGF20	605558	AR	Renal agenesis	[16]
FOXC1	601090	AD	Renal hypodysplasia	[17]
FRAS1	607830	AR	Fraser syndrome	[18,19]
FREM2	608945	AR	Fraser syndrome	[19,20]
GATA3	131320	AD	Hypoparathyroidism, sensorineural deafness, and renal disease (HDR) syndrome	[21]
GDF11	603936	Unknown	Candidate gene	[20]
GDNF	600837	AD	Renal agenesis	[22,23]
GFRA1	601496	AD	Renal agenesis	[23]
GREM1	603054	AR	Renal agenesis	[19]
HNF1B	189907	AD	Renal hypodysplasia, MCDK, and renal cyst	[24]
HOXC11	605559	Unknown	Renal agenesis and renal hypodysplasia in mice	[25]
HOXA11	142958	Unknown	Renal agenesis and renal hypodysplasia in mice	[25]
HOXD11	142986	Unknown	Renal agenesis and renal hypodysplasia in mice	[25]
KAL1	300836	XLR	Renal agenesis and Kallman syndrome	[26]
MMP-1	120353	AR	Obstructive uropathy	[27]
MMP-3	185250	Unknown	Obstructive uropathy	[27]
MMP-8	120355	Unknown	Obstructive uropathy	[27]
MUC1	158340	AD	Medullary cystic kidney disease type 1	[28]
NOTCH2	600275	AD	Alagille syndrome and Hajdu-Cheney syndrome with renal anomalies	[29,30]
PAX2	167409	AD	Renal coloboma syndrome and renal hypodysplasia	[31]
PKD1	601313	AD	Autosomal dominant polycystic kidney disease	[32]
PKD2	173910	AD	Autosomal dominant polycystic kidney disease	[32]

PKHD1	606702	AR	Autosomal recessive polycystic kidney disease	[33]
REN	179820	AR	Renal tubular dysgenesis	[1]
RET	164761	AD	Renal agenesis	[22]
ROBO2	602431	AD	VUR	[34,35]
SALL1	602218	AD	Renal hypodysplasia	[15]
SIX1	601205	AD	Branchio-oto-renal syndrome	[36]
SIX2	604994	AD	Renal hypodysplasia	[6]
SIX4	606342	Unknown	Candidate gene	[20]
SIX5	600963	AD	Branchio-oto-renal syndrome	[37]
SLIT2	603746	AD	Renal agenesis and MCDK	[38]
SOX17	610928	AD	VUR and UPJO	[39]
SPRY1	602465	Unknown	Candidate gene	[20]
TBX18	604613	AD	Urinary tract anomlies	[40]
TFAP2A	107580	AD	Branchio-oto-renal syndrome	[41]
TNXB	600985	AD	VUR	[42]
TRAP1	606219	AR	VUR, renal agenesis, and VACTERL association	[43]
TSC2	191092	AD	Cystic kidney disease in mice	[44]
UMOD	191845	AD	Medullary cystic kidney disease type 2 and hyperuricemic nephropathy	[45]
UPK3A	611559	AD	Renal adysplasia	[46]
USF2	600390	AD	Renal hypodysplasia	[47]
WNT4	603490	AD	Renal hypodysplasia	[48]
WT1	607102	AD	Diffuse mesangial sclerosis	[49]
XPNPEP3	613553	AR	Renal cysts and dysplasia	[50]

MIM, mendelian inheritance in man; AR, autosomal recessive; UPJO, ureteropelvic junction obstruction; AD, autosomal dominant; VUR,vesicoureteral reflux; MCDK, multicystic dysplastic kidney; XLR, X-linked recessive; VACTERL, vertebral defects, anal atresia, cardiac defects, trachea-esophageal fistula, renal anoamalies, and limb abnormalities.

S. Table 2. Single nucleotide variant prioritization and filtering workflow.

**Step**		**Process**	**Variants (average)**
1		Joint call variants	828,905
	1-1	Variants in coding region	18,398
	1-2	Not synonymous variants	12,078
	1-3	Rare variants (minor allele frequency < 1%) in 1000G, ExAC, and KRGDB	3,698
2		Sum of variants in each sample	5,285 (56.2)
	2-1	Variants in target 60 genes	620 (6.6)
	2-2	Qualified variants (Depth $\geq$ 5, VAF > 0.3, GQ $\geq$ 20)	358 (3.8)
3		Variant classification	
		Pathogenic	6
		Likely pathogenic	2
		Variants of uncertain significance	26
		Benign	324

1000G, 1000 Genomes Project; ExAC, Exome Aggregation Consortium; KRGDB, Korean Reference Genome Database; VAF, variant allele frequency; GQ, genotype quality.

S. Table 3. Variants of uncertain significance.

**Patient ID**	**Gene Transcript number**	**Nucleotide**	**Amino acid**	**Segregation**	**Frequency**		**MutationTaster**	**Renal phenotype**	Extra-renal phenotype	Renal function	Sanger seqeuncing
					**KRGDB**	**ExAC**					
3	*RET* NM_020975.6	c.1921G>A	p.Ala641Thr	NA	0.0014	0.00004	DC	Bilateral RHD, horseshoe kidney, and left VUR	Oligohydramnios	ESRD at 10.4 yr	Yes
8	*TNXB* NM_019105.8	c.9749C>T	p.Thr3250Met	NA	0	0.00006	DC	Left renal agenesis, right renal cysts, and right VUR	Testicular cystic mass and epidydimal cysts	CKD	No
8	*ROBO2* NM_002942.5	c.3553T>C	p.Trp1185Arg	NA	0	0	NA	Left renal agenesis, right renal cysts, and right VUR	Testicular cystic mass and epidydimal cysts	CKD	No
13	*TNXB* NM_019105.8	c.2090T>C	p.Leu697Pro	NA	0.0018	0.00006	DC	Right MCDK and left VUR	Imperforate anus, duodenal obstruction due to annular pancreas, and choledochal cyst	Normal at 9.8 yr	No
14	*RET* NM_020975.6	c.1618A>G	p.Arg540Gly	NA	0.0009	0.00004	DC	Bilateral RHD	Epilepsy, attention deficit hyperactivity disorder, and post-transplant diabetes mellitus	ESRD at 17.2 yr	No
16	*TNXB* NM_019105.8	c.1364G>A	p.Gly455Asp	NA	0.0009	0.00001	DC	Bilateral RHD	Birth asphyxia, nephrocalcinosis, hearing loss, periventricular leukomalasia, and cardiomyopathy	CKD	No
17	*RET* NM_020975.6	c.874G>A	p.Val292Met	Mother	0.0018	0.0006	DC	Right RHD	Hearing loss	CKD	Yes

20	*NOTCH2* NM_024408.4	c.5684G>A	p.Arg1895His	NA	0.0009	0.0001	DC	Left renal agenesis	Micropenis	CKD	Yes
22	*ROBO2* NM_002942.5	c.1435C>T	p.Arg479Trp	NA	0	0.00003	DC	Left RHD, right renal cysts, and bilateral VUR	Oligohydramnios, post-transplant diabetes mellitus	ESRD at 6.6 yr	No
28	*TNXB* NM_019105.8	c.2030A>G	p.Asp677Gly	NA	0.0045	0.0025	DC	Bilateral RHD and bilateral VUR	Preterm and oligohydroamnios	CKD	No
28	*NOTCH2* NM_024408.4	c.5557G>C	p.Asp1853His	NA	0	0.00002	DC	Bilateral RHD and bilateral VUR	Preterm and oligohydroamnios	CKD	No
30	*CHD1L* NM_004284.6	c.1841G>A	p.Arg614Gln	NA	0.0077	0.0001	DC	Bilateral RHD and renal cysts	Atrial septal defect, short stature, hearing loss, and hypothyroidim	ESRD at 2.9 yr	No
31	*CHD1L* NM_004284.6	c.2345T>C	p.Leu782Ser	NA	0.0036	0.00005	DC	Right renal agenesis and left hydronephrosis	Developmental delay, retroperitoneal pesudocyst, and hearing loss	CKD	No
41	*HNF1B* NM_000458.4	c.439C>G	p.Gln147Glu	NA	0	0	DC	Bilateral RHD and renal cysts	Patent ductus arteriosus, ureter stone, pancreas hypoplasia, common bile-duct dilatation, and hepatic cyst	CKD	Yes
55	*ROBO2* NM_002942.5	c.3585G>T	p.Gln1195His	NA	0	0.00001	NA	Bilateral VUR	No	CKD	Yes
58	*SLIT2* NM_004787.4	c.674G>A	p.Arg225His	NA	0.0005	0.0001	DC	Right MCDK and bilateral VUR	Parietal lobe epilepsy, multiple neuropathic pain, hypomagnesemia, and hyperuricemia	ESRD at 21.1 yr	No

63	*HNF1B* NM_000458.4	c.313G>A	p.Glu105Lys	NA	0.0014	0.0001	DC	Bilateral RHD and left VUR	No	ESRD at 20.1 yr	Yes
66	*SLIT2* NM_004787.4	c.1046C>T	p.Ser349Phe	NA	0.0005	0	DC	Bilateral VUR	No	CKD	No
73	*SIX2* NM_016932.5	c.707C>T	p.Pro236Leu	NA	0.0005	0.0002	DC	Bilateral RHD and bilateral VUR	Post-transplant diabetes mellitus	ESRD at 13.6 yr	No
73	*SLIT2* NM_004787.4	c.4488G>T	p.Arg1496Ser	NA	0.0014	0.00002	DC	Bilateral RHD and bilateral VUR	Post-transplant diabetes mellitus	ESRD at 13.6 yr	No
76	*TNXB* NM_019105.8	c.2030A>G	p.Asp677Gly	NA	0.0045	0.0025	DC	Right RHD and bilateral VUR	No	CKD	No
76	*NOTCH2* NM_024408.4	c.5557G>C	p.Asp1853His	NA	0	0.0002	DC	Right RHD and bilateral VUR	No	CKD	No
79	*DSTYK* NM_015375.3	c.1718T>C	p.Ile573Thr	NA	0	0	DC	Bilateral VUR	Complex febrile seizure and inguinal hernia	CKD	No
81	*ACE* NM_000789.4	c.2186G>A c.2803C>T	p.Arg729Gln p.Pro935Ser	NA	0.0009 0.0005	0.00003 0.00002	DC DC	Bilateral VUR	No	ESRD at 15.5 yr	Yes
86	*CHD1L* NM_004284.6	c.968A>T	p.Asp323Val	NA	0.0027	0.0001	DC	Right renal agenesis and left UVJO	Hematometra with upper vaginal obstruction	CKD	No

KRGDB, Korean Reference Genome Database; ExAC, Exome Aggregation Consortium; NA, not available; DC, disease causing; RHD, renal hypodysplasia; ESRD, end stage renal disease; CKD, chronic kidney disease; MCDK, multicystic dysplatic kidney; VUR, vesicoureteral refluex; UVJO, ureterovesical junction obstruction.

S. Table 4. Clinical presentation of study participants.

**Patient ID**	**Sex**	**Age at enrollment (yr)**	**Right kidney**	**Left kidney**	**Extra-renal phenotype**	**Renal outcome**	**Parental sampling**
1	F	21.1	Renal agenesis	Renal cortical cysts	No	ESRD at 20.8 yr	Father and mother
2	M	18.5	RHD	RHD	No	CKD stage 3 at 21.0 yr	ND
3	M	20.6	RHD	RHD and VUR	Oligohydroamnios	ESRD at 10.4 yr	NA

4	M	21.8	RHD	RHD	Preterm, oilgohydramnios, developmental delay, microcephaly, spastic quadriplesia, inguinal hernia, and post-transplant diabetes mellitus	ESRD at 6.8 yr	ND
5	F	19.4	RHD and UPJO	RHD and UPJO	No	ESRD at 18.7 yr	ND
6	F	19.3	No	Renal agenesis	No	Normal at 21.7 yr	ND
7	M	18.4	RHD	RHD	Morning glory discs, dilated cardiomyopathy, and hypothyrodism	ESRD at 10.4 yr	NA
8	M	17.0	Renal cortical cysts and VUR	Renal agenesis	Testicular cystic mass and epidydimal cysts	CKD stage 3 at 19.5 yr	NA
9	M	17.0	RHD	RHD and renal cortical cysts	No	ESRD at 16.9 yr	ND
10	M	17.3	No	RHD and VUR	Thyroid colloid cysts	CKD stage 2 at 20.1 yr	ND
11	M	20.0	RHD and renal cortical cysts	RHD and renal cortical cysts	Oligohydraminos	ESRD at 16.9 yr	ND
12	M	15.3	RHD	RHD	No	CKD stage 3 at 18.0 yr	ND
13	M	8.5	MCDK	VUR	Imperforate anus, duodenal obstruction due to annular pancreas, and choledochal cyst	Normal at 9.8 yr	NA
14	M	19.1	RHD	RHD	Epilepsy, attention deficit hyperactivity disorder, and post-transplant diabetes mellitus	ESRD at 17.2 yr	NA
15	F	14.5	RHD	RHD	Pre-auricular pit and hearing loss	ESRD at 5.4 yr	ND
16	M	16.3	RHD	RHD	Birth asphyxia, nephrocalcinosis, hearing loss, periventricular leukomalasia, and cardiomyopathy	CKD stage 5 at 17.3 yr	NA
17	F	17.6	RHD	No	Hearing loss	CKD stage 2 at 19.8 yr	Father and mother
18	M	0.8	RHD and VUR	RHD	Oligohydramnios	CKD stage 3 at 3.5 yr	NA
19	M	20.1	RHD	RHD	No	CKD stage 3 at 22.8 yr	NA
20	M	16.0	No	Renal agenesis	Micropenis	CKD stage 5 at 16.0 yr	NA
21	M	9.5	RHD	RHD	No	CKD stage 2 at 12.3 yr	NA

22	M	8.3	Renal cortical cysts and VUR	RHD and VUR	Oligohydramnios, post-transplant diabetes mellitus	ESRD at 6.6 yr	NA
23	M	9.1	RHD	RHD	Preterm, oilgohydramnios, and atrial septal defect	ESRD at 6.0 yr	NA
24	M	7.8	Renal agenesis	Duplicated ureter and hydronephrosis	Preterm, oilgohydramnios, tetralogy of Fallot, imperforate anus, anorectal malformation, patent urachus, strabismus, and inguinal hernia	CKD stage 2 at 10.2 yr	NA
25	F	13.7	RHD and renal cortical cysts	RHD and renal cortical cysts	Preterm and oligohydroamnios	ESRD at 7.4 yr	NA
26	M	6.1	RHD	RHD	Preterm and oligohydroamnios	CKD stage 3 at 8.9 yr	NA
28	M	5.9	RHD and VUR	RHD and VUR	Preterm and oligohydroamnios	CKD stage 3 at 8.7 yr	NA
29	M	5.3	RHD and renal cortical cysts	RHD and renal cortical cysts	Preterm, oliogohydroamnios, inguinal hernia, and developmental delay	ESRD at 1 mo	Father and mother
30	F	4.8	RHD and renal cortical cysts	RHD and renal cortical cysts	Atrial septal defect, short stature, hearing loss, and hypothyroidim	ESRD at 2.9 yr	NA
31	M	5.5	Renal agenesis	Hydronephrosis	Developmental delay, retroperitoneal pseudocyst, and hearing loss	CKD stage 3 at 8.3 yr	NA
32	M	8.3	RHD and VUR	VUR	No	CKD stage 2 at 11.2 yr	ND
33	M	15.9	RHD	Renal agenesis and VUR	No	CKD stage 5 at 18.7 yr	ND
34	M	12.2	RHD and renal cortical cysts	RHD and renal cortical cysts	Preterm, imperforate anus, hypospadias, and choledocal cyst	CKD 3 at 13.6 yr	ND
35	M	10.9	RHD	RHD	Preterm, oligohydroamios, branchial cleft cyst, lymphoma, developmental delay, autism, and inguinal hernia	ESRD at 12.1 yr	NA
36	M	4.6	RHD and renal cortical cysts	RHD and renal cortical cysts	Oligohydramnios, hereditary multiple exostoses and congenital ptosis	CKD stage 3 at 7.5 yr	ND
37	M	12.8	RHD and renal cortical cysts	RHD and renal cortical cysts	Preterm, oliogohydroamnios, attention deficit hyperactivity disorder	ESRD at 12.2 yr	ND
38	M	13.8	RHD and renal cortical cysts	RHD, renal cortical cysts, and VUR	Preterm, mental retardation, attention deficit hyperactivity disorder, hearing loss, and intermittent exotropia	CKD stage 3 at 16.6 yr	ND
39	F	22.1	RHD and renal cortical cysts	RHD and renal cortical cysts	No	ESRD at 13.4 yr	ND

40	M	11.2	RHD and renal cortical cysts	RHD and renal cortical cysts	Nail dysplasia	ESRD at 5.2 yr	ND
41	M	18.1	RHD and renal cortical cysts	RHD and renal cortical cysts	Patent ductus arteriosus, ureter stone, pancreas hypoplasia, common bile-duct dilatation, and hepatic cyst	CKD stage 5 at 20.3 yr	NA
42	M	9.8	RHD and renal cortical cysts	RHD and renal cortical cysts	Preterm, jeunal atresia, cerebral palsy (periventricular leukomalacia)	CKD stage 3 at 12.8 yr	ND
43	M	15.4	RHD and renal cortical cysts	RHD and renal cortical cysts	Hyperuricemia	CKD stage 3 at 15.8 yr	ND
44	M	24.5	RHD	Renal cortical cysts	Hyperuricemia	CKD stage 2 at 27 yr	ND
45	F	10.2	RHD and renal cortical cysts	RHD and renal cortical cysts	Preterm, oligohydraminios, hypothyroidism and Fanconi syndrome	CKD stage 5 at 13.2 yr	ND
46	M	23.2	RHD and renal cortical cysts	RHD	Thrombocytopenia	ESRD at 18.5 yr	ND
48	F	28.0	RHD	RHD	Hypothyroidism	ESRD at 27.9 yr	ND
49	M	1.6	VUR	RHD and VUR	Preterm and hypospadias	Normal at 4.4 yr	ND
50	M	26.1	Mid-ureteral obstruction	MCDK	Post-transplant diabetes mellitus	ESRD at 17.6 yr	ND
51	M	11.9	No	MCDK and ureterocele	Preterm, atrial septal defect, bilateral undescended testis, and omphalocele	Normal at 11.9 yr	ND
52	F	12.2	RHD and renal cortical cysts	MCDK	Preterm and oliogohydroamnios,	ESRD at 11 mo	ND
53	M	21.3	RHD	RHD and VUR	No	ESRD at 7.2 yr	ND
54	M	20.0	VUR	VUR	No	ESRD at 20.0 yr	ND
55	M	19.9	VUR	VUR	No	CKD stage 3 at 22.4 yr	NA
56	M	18.2	VUR	VUR	Intermittent exotropia	CKD stage 3 at 21.1 yr	ND
57	M	17.6	VUR	VUR	No	ESRD at 16.7 yr	ND
58	M	22.8	MCDK and VUR	VUR	Parietal lobe epilepsy, multiple neuropathic pain, hypomagnesemia, and hyperuricemia	ESRD at 21.1 yr	NA

59	M	16.8	VUR	VUR	No	CKD stage 5 at 19.8 yr	ND
60	M	17.3	RHD and VUR	RHD and VUR	No	ESRD at 17.0 yr	ND
61	M	19.3	VUR	VUR	Optic disc coloboma and strabismus	CKD stage 5 at 22.4 yr	ND
62	M	22.3	VUR	VUR	No	CKD stage 3 at 23.6 yr	ND
63	M	23.2	RHD	RHD and VUR	No	ESRD at 20.1 yr	NA
64	M	14.2	VUR	RHD and VUR	No	ESRD at 5.6 yr	ND
65	M	25.2	VUR	VUR	No	ESRD at 16.9 yr	ND
66	M	18.9	VUR	VUR	No	CKD stage 3 at 21.3 yr	NA
67	M	12.0	RHD and VUR	VUR	Atrial septal defect and epiblepharon	CKD stage 4 at 14.9 yr	ND
68	M	12.1	RHD and VUR	RHD and VUR	Oligohydraminos	ESRD at 8.9 yr	ND
69	M	21.6	RHD	RHD	No	ESRD at 20.0 yr	ND
70	M	21.2	RHD and VUR	VUR	Intellectual and developmental delay, nocturnal enuresis, and insomnia	CKD stage 4 at 21.2 yr	ND
71	M	9.0	VUR	VUR	Preterm	CKD stage 2 at 11.8 yr	ND
72	F	22.9	VUR	VUR	bilateral superior vena cava	ESRD at 21.6 yr	ND
73	M	12.9	RHD and VUR	RHD and VUR	Post-transplant diabetes mellitus	ESRD at 13.6 yr	NA
74	M	8.6	RHD	VUR	Preterm, umbilical hernia, inguinal hernia, and cryptorchidism	CKD stage 2 at 10.6 yr	ND
75	M	7.9	VUR	VUR	Preterm	CKD stage 4 at 10.8 yr	ND
76	M	19.7	RHD and VUR	VUR	No	CKD stage 2 at 19.7 yr	NA

77	M	11.9	RHD	RHD and VUR	No	CKD stage 4 at 11.9 yr	ND
78	M	4.9	RHD and VUR	VUR	No	ESRD at 4 mo	ND
79	M	15.5	VUR	VUR	Complex febrile seizure and inguinal hernia	CKD stage 5 at 18.2 yr	NA
80	M	9.3	RHD, renal cortical cysts, and VUR	RHD, renal cortical cysts, and VUR	Oligohydraminos, developmental delay and sensory neural hearing loss	ESRD at 2 mo	ND
81	M	15.8	VUR	VUR	No	ESRD at 15.5 yr	NA
82	M	15.5	RHD and renal cortical cysts	RHD and renal cortical cysts	Diabetes mellitus	Normal at 18.1 yr	ND
83	M	16.9	PUV	PUV	Hearing loss	CKD stage 3 at 19.8 yr	ND
84	M	5.3	PUV and RHD	PUV and RHD	Preterm and oliogohydroamnios	CKD stage 4 at 8.2 yr	ND
85	M	4.3	PUV and RHD	PUV and RHD	Preterm, oliogohydroamnios, hypothyrodism, inguinal hernia, hypertrophic cardiomyopathy, auricular benign cyst, and developmental delay	ESRD at 5 mo	ND
86	F	20.6	Renal agenesis	UVJO	Hematometra with upper vaginal obstruction	CKD stage 2 at 23.6 yr	NA
87	M	13.8	UPJO	UPJO	No	ESRD at 13.4 yr	ND
88	F	3.1	VUR	Renal agenesis	No	CKD stage 2 at 5.8 yr	ND
89	M	14.7	Hydronephrosis	MCDK	No	CKD stage 2 at 5.1 yr	ND
90	F	14.4	UPJO	MCDK	Partial bicornuate uterus or partial septated uterus	CKD stage 2 at 15.4 yr	ND
91	F	10.7	MCDK	Hydronephrosis	Preterm, oliogohydroamnios, imperforated anus, vaginal anomaly, and ambiguous genitalia	CKD stage 2 at 13.1 yr	ND
92	M	10.4	RHD and renal cortical cysts	MCDK	Oligohydraminos, choledochal cyst, and hypomagnesemia	CKD stage 2 at 13.0 yr	Father and mother
93	M	3.5	Renal cortical cysts	MCDK	Preterm and portal vein thrombosis	Normal at 3.5 yr	ND

94	M	15.6	RHD and renal cortical cysts	RHD and renal cortical cysts	Developmental delay, Atrial septal defect, cleft palate, cerebral palsy, congenital patellar dislocation, congenital blepharoptosis, and skeletal dysplasia	CKD sate 2 at 18.1 yr	ND
95	M	14.5	RHD and renal cortical cysts	RHD and renal cortical cysts	Optic disc anomaly, hemolytic anemia, and attention deficit hyperactivity disorder	ESRD at 3.2 yr	Father and mother
96	M	10.9	VUR and ureterocele	RHD	No	CKD stage 3 at 13.9 yr	ND

Yr, years; F, female; M, male; ESRD, end stage renal disease; RHD, renal hypodysplasia; CKD, chronic kidney disease; ND, not done; NA, not available; VUR, vesicoureteral reflux; UPJO, ureteropelvic junction obstruction; MCDK, multicystic dysplastic kidney; mo, monthrs; UVJO, ureterovesical junction obstruction; PUV, posterior urethral valve.

References

1. Gribouval, O.; Moriniere, V.; Pawtowski, A.; Arrondel, C.; Sallinen, S.L.; Saloranta, C.; Clericuzio, C.; Viot, G.; Tantau, J.; Blesson, S., et al. Spectrum of mutations in the renin-angiotensin system genes in autosomal recessive renal tubular dysgenesis. Human mutation **2012**, *33*, 316-326, doi:10.1002/humu.21661.
2. Oh, S.P.; Li, E. The signaling pathway mediated by the type IIB activin receptor controls axial patterning and lateral asymmetry in the mouse. Genes Dev **1997**, *11*, 1812-1826, doi:10.1101/gad.11.14.1812.
3. Weber, S.; Landwehr, C.; Renkert, M.; Hoischen, A.; Wuhl, E.; Denecke, J.; Radlwimmer, B.; Haffner, D.; Schaefer, F.; Weber, R.G. Mapping candidate regions and genes for congenital anomalies of the kidneys and urinary tract (CAKUT) by array-based comparative genomic hybridization. Nephrology, dialysis, transplantation: Official publication of the European Dialysis and Transplant Association - European Renal Association **2011**, *26*, 136-143, doi:10.1093/ndt/gfq400.
4. Nishimura, H.; Yerkes, E.; Hohenfellner, K.; Miyazaki, Y.; Ma, J.; Hunley, T.E.; Yoshida, H.; Ichiki, T.; Threadgill, D.; Phillips, J.A., 3rd, et al. Role of the angiotensin type 2 receptor gene in congenital anomalies of the kidney and urinary tract, CAKUT, of mice and men. Molecular cell **1999**, *3*, 1-10.
5. Kraus, M.R.; Clauin, S.; Pfister, Y.; Di Maio, M.; Ulinski, T.; Constam, D.; Bellanne-Chantelot, C.; Grapin-Botton, A. Two mutations in human BICC1 resulting in Wnt pathway hyperactivity associated with cystic renal dysplasia. Human mutation **2012**, *33*, 86-90, doi:10.1002/humu.21610.
6. Weber, S.; Taylor, J.C.; Winyard, P.; Baker, K.F.; Sullivan-Brown, J.; Schild, R.; Knuppel, T.; Zurowska, A.M.; Caldas-Alfonso, A.; Litwin, M., et al. SIX2 and BMP4 mutations associate with anomalous kidney development. Journal of the American Society of Nephrology: JASN **2008**, *19*, 891-903, doi:10.1681/ASN.2006111282.
7. Dudley, A.T.; Lyons, K.M.; Robertson, E.J. A requirement for bone morphogenetic protein-7 during development of the mammalian kidney and eye. Genes Dev **1995**, *9*, 2795-2807.
8. Hwang, D.Y.; Dworschak, G.C.; Kohl, S.; Saisawat, P.; Vivante, A.; Hilger, A.C.; Reutter, H.M.; Soliman, N.A.; Bogdanovic, R.; Kehinde, E.O., et al. Mutations in 12 known dominant disease-causing genes clarify many congenital anomalies of the kidney and urinary tract. Kidney international **2014**, *85*, 1429-1433, doi:10.1038/ki.2013.508.
9. Groenen, P.M.; Vanderlinden, G.; Devriendt, K.; Fryns, J.P.; Van de Ven, W.J. Rearrangement of the human CDC5L gene by a t(6;19)(p21;q13.1) in a patient with multicystic renal dysplasia. Genomics **1998**, *49*, 218-229, doi:10.1006/geno.1998.5254.
10. Brockschmidt, A.; Chung, B.; Weber, S.; Fischer, D.C.; Kolatsi-Joannou, M.; Christ, L.; Heimbach, A.; Shtiza, D.; Klaus, G.; Simonetti, G.D., et al. CHD1L: A new candidate gene for congenital anomalies of the kidneys and urinary tract (CAKUT). Nephrology, dialysis, transplantation: Official publication of the European Dialysis and Transplant Association - European Renal Association **2012**, *27*, 2355-2364, doi:10.1093/ndt/gfr649.
11. Suzuki, K.; Haraguchi, R.; Ogata, T.; Barbieri, O.; Alegria, O.; Vieux-Rochas, M.; Nakagata, N.; Ito, M.; Mills, A.A.; Kurita, T., et al. Abnormal urethra formation in mouse models of split-hand/split-foot malformation type 1 and type 4. European journal of human genetics: EJHG **2008**, *16*, 36-44, doi:10.1038/sj.ejhg.5201925.
12. Sanna-Cherchi, S.; Sampogna, R.V.; Papeta, N.; Burgess, K.E.; Nees, S.N.; Perry, B.J.; Choi, M.; Bodria, M.; Liu, Y.; Weng, P.L., et al. Mutations in DSTYK and dominant urinary tract malformations. The New England journal of medicine **2013**, *369*, 621-629, doi:10.1056/NEJMoa1214479.
13. Boualia, S.K.; Gaitan, Y.; Murawski, I.; Nadon, R.; Gupta, I.R.; Bouchard, M. Vesicoureteral reflux and other urinary tract malformations in mice compound heterozygous for Pax2 and Emx2. *PLoS ONE* 2011, 6, e21529, doi:10.1371/journal.pone.0021529.
14. Orten, D.J.; Fischer, S.M.; Sorensen, J.L.; Radhakrishna, U.; Cremers, C.W.; Marres, H.A.; Van Camp, G.; Welch, K.O.; Smith, R.J.; Kimberling, W.J. Branchio-oto-renal syndrome (BOR): Novel mutations in the EYA1 gene, and a review of the mutational genetics of BOR. Human mutation **2008**, *29*, 537-544, doi:10.1002/humu.20691.
15. Weber, S.; Moriniere, V.; Knuppel, T.; Charbit, M.; Dusek, J.; Ghiggeri, G.M.; Jankauskiene, A.; Mir, S.; Montini, G.; Peco-Antic, A., et al. Prevalence of mutations in renal developmental genes in children with renal hypodysplasia: Results of the ESCAPE study. Journal of the American Society of Nephrology: JASN **2006**, *17*, 2864-2870, doi:10.1681/ASN.2006030277.

16. Barak, H.; Huh, S.H.; Chen, S.; Jeanpierre, C.; Martinovic, J.; Parisot, M.; Bole-Feysot, C.; Nitschke, P.; Salomon, R.; Antignac, C., et al. FGF9 and FGF20 maintain the stemness of nephron progenitors in mice and man. Dev Cell **2012**, *22*, 1191-1207, doi:10.1016/j.devcel.2012.04.018.
17. Nakano, T.; Niimura, F.; Hohenfellner, K.; Miyakita, E.; Ichikawa, I. Screening for mutations in BMP4 and FOXC1 genes in congenital anomalies of the kidney and urinary tract in humans. The Tokai journal of experimental and clinical medicine **2003**, *28*, 121-126.
18. van Haelst, M.M.; Maiburg, M.; Baujat, G.; Jadeja, S.; Monti, E.; Bland, E.; Pearce, K.; Fraser Syndrome Collaboration, G.; Hennekam, R.C.; Scambler, P.J. Molecular study of 33 families with Fraser syndrome new data and mutation review. American journal of medical genetics. Part A 2008, 146A, 2252-2257, doi:10.1002/ajmg.a.32440.
19. Kohl, S.; Hwang, D.Y.; Dworschak, G.C.; Hilger, A.C.; Saisawat, P.; Vivante, A.; Stajic, N.; Bogdanovic, R.; Reutter, H.M.; Kehinde, E.O., et al. Mild recessive mutations in six Fraser syndrome-related genes cause isolated congenital anomalies of the kidney and urinary tract. Journal of the American Society of Nephrology: JASN **2014**, *25*, 1917-1922, doi:10.1681/ASN.2013101103.
20. Saisawat, P.; Tasic, V.; Vega-Warner, V.; Kehinde, E.O.; Gunther, B.; Airik, R.; Innis, J.W.; Hoskins, B.E.; Hoefele, J.; Otto, E.A., et al. Identification of two novel CAKUT-causing genes by massively parallel exon resequencing of candidate genes in patients with unilateral renal agenesis. Kidney international **2012**, *81*, 196-200, doi:10.1038/ki.2011.315.
21. Ali, A.; Christie, P.T.; Grigorieva, I.V.; Harding, B.; Van Esch, H.; Ahmed, S.F.; Bitner-Glindzicz, M.; Blind, E.; Bloch, C.; Christin, P., et al. Functional characterization of GATA3 mutations causing the hypoparathyroidism-deafness-renal (HDR) dysplasia syndrome: Insight into mechanisms of DNA binding by the GATA3 transcription factor. Human molecular genetics **2007**, *16*, 265-275, doi:10.1093/hmg/ddl454.
22. Skinner, M.A.; Safford, S.D.; Reeves, J.G.; Jackson, M.E.; Freemerman, A.J. Renal aplasia in humans is associated with RET mutations. American journal of human genetics **2008**, *82*, 344-351, doi:10.1016/j.ajhg.2007.10.008.
23. Chatterjee, R.; Ramos, E.; Hoffman, M.; VanWinkle, J.; Martin, D.R.; Davis, T.K.; Hoshi, M.; Hmiel, S.P.; Beck, A.; Hruska, K., et al. Traditional and targeted exome sequencing reveals common, rare and novel functional deleterious variants in RET-signaling complex in a cohort of living US patients with urinary tract malformations. Human genetics **2012**, *131*, 1725-1738, doi:10.1007/s00439-012-1181-3.
24. Clissold, R.L.; Hamilton, A.J.; Hattersley, A.T.; Ellard, S.; Bingham, C. HNF1B-associated renal and extra-renal disease-an expanding clinical spectrum. Nat Rev Nephrol **2015**, *11*, 102-112, doi:10.1038/nrneph.2014.232.
25. Wellik, D.M.; Hawkes, P.J.; Capecchi, M.R. Hox11 paralogous genes are essential for metanephric kidney induction. Genes Dev **2002**, *16*, 1423-1432, doi:10.1101/gad.993302.
26. Albuisson, J.; Pecheux, C.; Carel, J.C.; Lacombe, D.; Leheup, B.; Lapuzina, P.; Bouchard, P.; Legius, E.; Matthijs, G.; Wasniewska, M., et al. Kallmann syndrome: 14 novel mutations in KAL1 and FGFR1 (KAL2). Human mutation **2005**, *25*, 98-99, doi:10.1002/humu.9298.
27. Djuric, T.; Zivkovic, M.; Milosevic, B.; Andjelevski, M.; Cvetkovic, M.; Kostic, M.; Stankovic, A. MMP-1 and -3 haplotype is associated with congenital anomalies of the kidney and urinary tract. Pediatric nephrology **2014**, *29*, 879-884, doi:10.1007/s00467-013-2699-x.
28. Kirby, A.; Gnirke, A.; Jaffe, D.B.; Baresova, V.; Pochet, N.; Blumenstiel, B.; Ye, C.; Aird, D.; Stevens, C.; Robinson, J.T., et al. Mutations causing medullary cystic kidney disease type 1 lie in a large VNTR in MUC1 missed by massively parallel sequencing. Nature genetics **2013**, *45*, 299-303, doi:10.1038/ng.2543.
29. Kamath, B.M.; Bauer, R.C.; Loomes, K.M.; Chao, G.; Gerfen, J.; Hutchinson, A.; Hardikar, W.; Hirschfield, G.; Jara, P.; Krantz, I.D., et al. NOTCH2 mutations in Alagille syndrome. Journal of medical genetics **2012**, *49*, 138-144, doi:10.1136/jmedgenet-2011-100544.
30. Narumi, Y.; Min, B.J.; Shimizu, K.; Kazukawa, I.; Sameshima, K.; Nakamura, K.; Kosho, T.; Rhee, Y.; Chung, Y.S.; Kim, O.H., et al. Clinical consequences in truncating mutations in exon 34 of NOTCH2: Report of six patients with Hajdu-Cheney syndrome and a patient with serpentine fibula polycystic kidney syndrome. American journal of medical genetics. Part A 2013, 161A, 518-526, doi:10.1002/ajmg.a.35772.
31. Bower, M.; Salomon, R.; Allanson, J.; Antignac, C.; Benedicenti, F.; Benetti, E.; Binenbaum, G.; Jensen, U.B.; Cochat, P.; DeCramer, S., et al. Update of PAX2 mutations in renal coloboma syndrome and establishment of a locus-specific database. Human mutation **2012**, *33*, 457-466, doi:10.1002/humu.22020.
32. Rossetti, S.; Consugar, M.B.; Chapman, A.B.; Torres, V.E.; Guay-Woodford, L.M.; Grantham, J.J.; Bennett, W.M.; Meyers, C.M.; Walker, D.L.; Bae, K., et al. Comprehensive molecular diagnostics in autosomal

dominant polycystic kidney disease. Journal of the American Society of Nephrology: JASN **2007**, *18*, 2143-2160, doi:10.1681/ASN.2006121387.

33. Ward, C.J.; Hogan, M.C.; Rossetti, S.; Walker, D.; Sneddon, T.; Wang, X.; Kubly, V.; Cunningham, J.M.; Bacallao, R.; Ishibashi, M., et al. The gene mutated in autosomal recessive polycystic kidney disease encodes a large, receptor-like protein. Nature genetics **2002**, *30*, 259-269, doi:10.1038/ng833.
34. Bertoli-Avella, A.M.; Conte, M.L.; Punzo, F.; de Graaf, B.M.; Lama, G.; La Manna, A.; Polito, C.; Grassia, C.; Nobili, B.; Rambaldi, P.F., et al. ROBO2 gene variants are associated with familial vesicoureteral reflux. Journal of the American Society of Nephrology: JASN **2008**, *19*, 825-831, doi:10.1681/ASN.2007060692.
35. Lu, W.; van Eerde, A.M.; Fan, X.; Quintero-Rivera, F.; Kulkarni, S.; Ferguson, H.; Kim, H.G.; Fan, Y.; Xi, Q.; Li, Q.G., et al. Disruption of ROBO2 is associated with urinary tract anomalies and confers risk of vesicoureteral reflux. American journal of human genetics **2007**, *80*, 616-632, doi:10.1086/512735.
36. Krug, P.; Moriniere, V.; Marlin, S.; Koubi, V.; Gabriel, H.D.; Colin, E.; Bonneau, D.; Salomon, R.; Antignac, C.; Heidet, L. Mutation screening of the EYA1, SIX1, and SIX5 genes in a large cohort of patients harboring branchio-oto-renal syndrome calls into question the pathogenic role of SIX5 mutations. Human mutation **2011**, *32*, 183-190, doi:10.1002/humu.21402.
37. Hoskins, B.E.; Cramer, C.H.; Silvius, D.; Zou, D.; Raymond, R.M.; Orten, D.J.; Kimberling, W.J.; Smith, R.J.; Weil, D.; Petit, C., et al. Transcription factor SIX5 is mutated in patients with branchio-oto-renal syndrome. American journal of human genetics **2007**, *80*, 800-804, doi:10.1086/513322.
38. Hwang, D.Y.; Kohl, S.; Fan, X.; Vivante, A.; Chan, S.; Dworschak, G.C.; Schulz, J.; van Eerde, A.M.; Hilger, A.C.; Gee, H.Y., et al. Mutations of the SLIT2-ROBO2 pathway genes SLIT2 and SRGAP1 confer risk for congenital anomalies of the kidney and urinary tract. Human genetics **2015**, *134*, 905-916, doi:10.1007/s00439-015-1570-5.
39. Gimelli, S.; Caridi, G.; Beri, S.; McCracken, K.; Bocciardi, R.; Zordan, P.; Dagnino, M.; Fiorio, P.; Murer, L.; Benetti, E., et al. Mutations in SOX17 are associated with congenital anomalies of the kidney and the urinary tract. Human mutation **2010**, *31*, 1352-1359, doi:10.1002/humu.21378.
40. Vivante, A.; Kleppa, M.J.; Schulz, J.; Kohl, S.; Sharma, A.; Chen, J.; Shril, S.; Hwang, D.Y.; Weiss, A.C.; Kaminski, M.M., et al. Mutations in TBX18 Cause Dominant Urinary Tract Malformations via Transcriptional Dysregulation of Ureter Development. American journal of human genetics **2015**, *97*, 291-301, doi:10.1016/j.ajhg.2015.07.001.
41. Li, H.; Sheridan, R.; Williams, T. Analysis of TFAP2A mutations in Branchio-Oculo-Facial Syndrome indicates functional complexity within the AP-2alpha DNA-binding domain. Human molecular genetics **2013**, *22*, 3195-3206, doi:10.1093/hmg/ddt173.
42. Gbadegesin, R.A.; Brophy, P.D.; Adeyemo, A.; Hall, G.; Gupta, I.R.; Hains, D.; Bartkowiak, B.; Rabinovich, C.E.; Chandrasekharappa, S.; Homstad, A., et al. TNXB mutations can cause vesicoureteral reflux. Journal of the American Society of Nephrology: JASN **2013**, *24*, 1313-1322, doi:10.1681/ASN.2012121148.
43. Saisawat, P.; Kohl, S.; Hilger, A.C.; Hwang, D.Y.; Yung Gee, H.; Dworschak, G.C.; Tasic, V.; Pennimpede, T.; Natarajan, S.; Sperry, E., et al. Whole-exome resequencing reveals recessive mutations in TRAP1 in individuals with CAKUT and VACTERL association. Kidney international **2014**, *85*, 1310-1317, doi:10.1038/ki.2013.417.
44. Hernandez, O.; Way, S.; McKenna, J., 3rd; Gambello, M.J. Generation of a conditional disruption of the Tsc2 gene. Genesis **2007**, *45*, 101-106, doi:10.1002/dvg.20271.
45. Hart, T.C.; Gorry, M.C.; Hart, P.S.; Woodard, A.S.; Shihabi, Z.; Sandhu, J.; Shirts, B.; Xu, L.; Zhu, H.; Barmada, M.M., et al. Mutations of the UMOD gene are responsible for medullary cystic kidney disease 2 and familial juvenile hyperuricaemic nephropathy. Journal of medical genetics **2002**, *39*, 882-892.
46. Jenkins, D.; Bitner-Glindzicz, M.; Malcolm, S.; Hu, C.C.; Allison, J.; Winyard, P.J.; Gullett, A.M.; Thomas, D.F.; Belk, R.A.; Feather, S.A., et al. De novo Uroplakin IIIa heterozygous mutations cause human renal adysplasia leading to severe kidney failure. Journal of the American Society of Nephrology: JASN **2005**, *16*, 2141-2149, doi:10.1681/ASN.2004090776.
47. Groenen, P.M.; Garcia, E.; Debeer, P.; Devriendt, K.; Fryns, J.P.; Van de Ven, W.J. Structure, sequence, and chromosome 19 localization of human USF2 and its rearrangement in a patient with multicystic renal dysplasia. Genomics **1996**, *38*, 141-148, doi:10.1006/geno.1996.0609.
48. Vivante, A.; Mark-Danieli, M.; Davidovits, M.; Harari-Steinberg, O.; Omer, D.; Gnatek, Y.; Cleper, R.; Landau, D.; Kovalski, Y.; Weissman, I., et al. Renal hypodysplasia associates with a WNT4 variant that causes aberrant canonical WNT signaling. Journal of the American Society of Nephrology: JASN **2013**, *24*, 550-558, doi:10.1681/ASN.2012010097.

49. Jeanpierre, C.; Denamur, E.; Henry, I.; Cabanis, M.O.; Luce, S.; Cecille, A.; Elion, J.; Peuchmaur, M.; Loirat, C.; Niaudet, P., et al. Identification of constitutional WT1 mutations, in patients with isolated diffuse mesangial sclerosis, and analysis of genotype/phenotype correlations by use of a computerized mutation database. American journal of human genetics **1998**, *62*, 824-833, doi:10.1086/301806.50.
50. O'Toole, J.F.; Liu, Y.; Davis, E.E.; Westlake, C.J.; Attanasio, M.; Otto, E.A.; Seelow, D.; Nurnberg, G.; Becker, C.; Nuutinen, M., et al. Individuals with mutations in XPNPEP3, which encodes a mitochondrial protein, develop a nephronophthisis-like nephropathy. The Journal of clinical investigation **2010**, *120*, 791-802, doi:10.1172/JCI40076.
